# Identification of the Interaction between P-Glycoprotein and Anxa2 in Multidrug-resistant Human Breast Cancer Cells

**DOI:** 10.3969/j.issn.2095-3941.2012.02.003

**Published:** 2012-06

**Authors:** Hai-chang Zhang, Fei Zhang, Bing Wu, Jing-hua Han, Wei Ji, Yan Zhou, Rui-fang Niu

**Affiliations:** Public Laboratory, Key Laboratory of Breast Cancer Prevention and Therapy, Tianjin Medical University, Ministry of Education, Tianjin Medical University Cancer Institute and Hospital, Tianjin 300060, China

**Keywords:** P-glycoprotein, Anxa2, drug resistance, multiple, neoplasm metastasis, breast neoplasm

## Abstract

**Objective:**

To explore the interaction of Anxa2 with P-Glycoprotein (P-gp) in the migration and invasion of the multidrug-resistant (MDR) human breast cancer cell line MCF-7/ADR.

**Methods:**

A pair of short hairpin RNA (shRNA) targeting P-gp was transfected into MCF-7/ADR cells, and monoclonal cell strains were screened. The expression of P-gp was detected by Western blot. Transwell chambers were used to observe the cell migration capacity and invasion ability. The interaction between P-gp and Anxa2 was examined by immunoprecipitation and immunofluorescence confocal microscopy analyses.

**Results:**

P-gp expression was significantly knocked down, and there were notable decreasing trends in the migration and invasion capability of MDR breast cancer cells (*P*<0.05). There was a close interaction between Anxa2 and P-gp.

**Conclusions:**

MCF-7/ADR is an MDR human breast cancer cell line with high migration and invasion abilities. The knockdown of P-gp notably impaired the migration and invasion abilities of the tumor cells. The interaction of Anxa2 with P-pg may play an important role in the enhanced invasiveness of MDR human breast cancer cells.

## Introduction

Breast cancer is a malignant tumor that can seriously affect women’s health. In recent years, the two leading causes of failure of the clinical treatment for cancer have been the development of metastases and drug resistance. These two important, but not clearly related aspects in the development of cancer have been extensively studied. The extracellular matrix metalloproteinase inducer, a cell membrane glycoprotein involved in metastases, is reportedly over-expressed in multidrug-resistant cells but not in drug-sensitive parental cell lines ^[^^1^^-^^3^^]^. Miletti-González et al. ^[^^4^^, ^^5^^]^ reported that the functional interaction between CD44s and P-glycoprotein (P-gp) was one step in a complex molecular organization that results in the concomitant multidrug resistance (MDR) phenotype as well as increased cell migration, *in vitro* invasion, and metastasis. The knockdown of MDR1 by short hairpin RNA (shRNA) impairs the migration and invasion abilities of tumor cells. Most MDR cases are widely accepted to be due to the over-expression of P-gp, a 170 kDa plasma membrane ATPase ^[^^6^^]^. Zhang et al.^[^^7^^]^ also showed that Anxa2 was up-regulated in multidrug-resistant MCF-7/ADR cells and might play essential roles in modulating MDR-induced tumor invasion/metastasis. P-gp and Anxa2 are known to be determinants of MDR and metastases. However, few reports have linked the two phenotypes, and reasons to suspect a direct connection have only been given recently. This study explored the interaction of Anxa2 with P-Glycoprotein (P-gp) in the migration and invasion of the multidrug-resistant human breast cancer cell line MCF-7/ADR.

## Materials and Methods

### Cell culture

Human breast cancer MCF-7 cells and the corresponding multidrug-resistant variant (adriamycin- resistant) MCF-7/ADR cells were provided by Dr. Zi-zheng Hou of the Detroit Hospital, Detroit, MI, USA. The cells were cultured in RPMI-1640 medium supplemented with 10% fetal bovine serum (Hyclone, USA) at 37°C under a humidified 5% CO_2_ atmosphere. The MCF-7/ADR cells were incubated in the presence of 0.5 µM adriamycin to maintain the MDR1 expression level. The cells were transferred to a drug-free medium for at least 1 month prior to experiments.

### shRNA design, construction, and transfection

The shRNA expression vector pGCsilencer U6/hygro was purchased from Genechem (Shanghai, China). The targeting sequence 5′-GGATGTGAGTTGGTTTGAT-3′ against MDR1 mRNA was designed, and the specificity was confirmed using a BLAST search against the human genome. ShRNA with a scrambled sequence (5′-TTCTCCGAACGTGTCACGT-3) was used as a negative control (Shanghai Gene Chem Co., Ltd). MCF-7/ADR cells were transfected with shMDR1 using Lipofectamine 2000 (Invitrogen, Carlsbad, CA, USA) according to the manufacturer’s instructions. Three independent MCF-7/ADR stable transfectants (named shMDR1/MCF-7/ADR) were screened for low P-gp expression after 2 weeks of selection in 300 µg/mL hygromycin. All transfectants were routinely cultured in a selection medium.

### Western blot and co-immunoprecipitation

Total proteins were extracted using RIPA lysis buffer. About 50 µg of lysates per sample was separated by SDS-PAGE using 10% polyacrylamide gels, and then transferred onto a PVDF membrane. The membrane was blocked with 5% skim milk, followed by incubation at 4°C overnight with the following primary antibodies: against P-gp (H-241, 1:500, Santa Cruz Biotechnology, CA, USA), Anxa2 (C10, 1:5000, Santa Cruz Biotechnology, CA, USA), and β-actin (1:5000, Santa Cruz Biotechnology, CA, USA). The corresponding proteins were immunodetected by incubating with the appropriate IRDye680/800nm-conjugated secondary antibodies (1:5000, Li-Cor BioSciences, Lincoln, NE, USA). The signals were analyzed using the Odyssey InfraRed Imaging System (Li-Cor BioSciences, Lincoln, NE, USA). For the co-immunoprecipitation assay, MCF-7/ADR cells were lysed in ice-cold lysis RIPA buffer [150 mM NaCl, pH 7.4 50 mM Tris–HCl, 5 mM EDTA, 1% Triton X-100, 0.1% SDS, 0.1 mM PMSF, and 20 mM Phosphatase Inhibitor Cocktail Tablets (Roche Diagnostics GmbH, Germany)]. About 1 mg of total protein lysate was then separately immunoprecipitated with 2 µg of anti-P-gp (JSB-1) and anti-Anxa2 (C10) antibodies overnight at 4°C in the presence of 50 µL of protein G-Sepharose beads (Millipore Chemicon, Becton Dickinson, USA). Normal mouse IgG antibody (Santa Cruz Biotechnology) was applied as an internal control. Immunoblotting detections were completed as described above.

### Adriamycin sensitivity assay

The colorimetric 3-(4,5-Dimethylthiazol-2-yl)-2,5-diphenyltetrazolium bromide (MTT)-based cytotoxicity assay was used to determine cell sensitivity toward adriamycin. About 5 × 10^3^ cells were cultured in 96-well plates, and different concentrations of adriamycin (0, 1, 5, 10, 20, 50, and 100 µmol/L) were added 24 h later. After 72 h incubation, 20 µL of 5 mg/mL MTT solution (Pu Boxin Biotechnology Co., Ltd., Beijing, China) was added into each well, which were then incubated for 4 h at 37°C. The MTT-formazan product dissolved in 150 µL of DMSO (Sigma-Aldrich) was evaluated by measuring its absorbance at 570 nm using a micro-plate reader (Tecan-5082 Sunrise, Australia). The experiment was repeated twice in triplicate.

### Tumor cell invasion and migration assays

Cell migration was measured in 12-well Boyden chamber plates with 8 µm-pore size polycarbonate membrane filter inserts (Millipore Chemicon, Becton Dickinson, USA). For the cell invasion assay, the interior of the transwell inserts was coated with diluted matrigel (BECTON DICKINSON, USA) that imitates the basement membrane. A total of 1 × 10^5^ cells were seeded onto the upper chamber, the cell suspension was seeded onto the membrane in the upper chamber, and the lower chamber was filled with 1200 µL of 10% FBS-DMEM. After incubation for 48 h, non-migrating cells in the upper chamber surface were removed with cotton swabs. The migrating cells at the bottom side of the membrane were fixed with formaldehyde for 10 min and then stained with a 3-Step Stain Set Kit (Richard-Allen Scientific, Kalamazoo, MI, USA) according to the manufacturer’s instructions. The stained membranes were cut and placed onto a glass slide, and the number of invading cells at the bottom surface of the membrane was counted under a bright field light microscope. All assays were performed in triplicate.

### Confocal immunofluorescence microscopy analysis

Confocal immunofluorescence microscopy imaging was used to detect the sub-cellular localization of Anxa2 and P-gp in MCF-7/ADR cells. The cells were grown on glass slides in 12-well plates. The slides were quickly and gently washed twice with PBS, followed by fixation in 100% methanol at -20°C for 10 min. Then, they were treated with 25 mM NH_4_Cl in PBS for 10 min to quench the free aldehyde groups, and permeabilized by incubation in freshly prepared 0.05% Saponin (Sigma) for 15 min. After 3 × PBS washes, the cells were pre-incubated with a blocking solution containing 3% normal goat serum for 2 h at room temperature, and incubated with the same blocking solution containing primary antibodies against P-gp (H-241, 1:50) or Anxa2 (C10, 1:200) overnight in a humid chamber at 4°C. After removal of the primary antibodies with 3 × PBS washes, the appropriate secondary antibodies (Alexa Flour488 goat anti-mouse IgG or Alexa Flour 546 goat anti-rabbit IgG, 1:200; Invitrogen) were incubated on the slides for 2 h at room temperature. The slides were mounted using Vectashield mounting medium (Vector Labs), and the cell nuclei were counterstained with DAPI for 10 min. Signals were visualized under a Leica TCS SP5 CLSM microscope (Heidelberg, Germany) with filters for the corresponding fluorescent stains.

### Wound-healing assay

The cells were cultured on glass slides in 12-well plates until 100% confluency. A sterile pipette tip was used to make a straight line wound on the confluent cells. After washing thrice with PBS, the remaining cells were further cultured for 48 h in a 0.5% bovine serum albumin (BSA)-DMEM medium. At the indicated time points, the sub-cellular localization of Anxa2 and P-gp was detected as described above.

### Statistical analysis

All data quantification and statistical analyses were performed using SPSS 13.0 software. A comparison among different experimental groups was performed using one-way ANOVA or Student’s *t*-test, when appropriate. Data were presented as the mean ± SD, and all experiments were independently repeated at least three times. *P* values <0.05 (two-sided) were considered statistically significant.

## Results

### Screening of stable clones down-expressing P-gp

**Figure 1:**
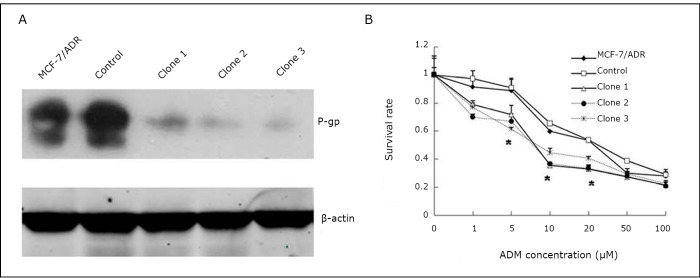
Screening of stable clones down-expressing P-gp. A: Western blot analysis of P-gp expression in MCF-7/ADR cells, 3 stable clones of shMDR1/MCF-7/ADR cells and control cells (transfected with a shRNA plasmid containing scrambled targeting sequence). P-gp expression was successfully inhibited in the knockdown experiments. B: The 3 P-gp knockdown clones were signiﬁcantly more sensitive to adriamycin treatment than the MCF-7/ADR wild-type and control cells. The experiments were repeated at least 3 times. The statistical significance was assessed by one-way ANOVA, *P<0.05 *vs.* control.

Three stable transfectants of shMDR1/MCF-7/ADR cells were screened and maintained. As shown in **Figure 1A**, Western blot analysis was used to screen the stable clones for knocking down P-gp expression. The P-gp expression levels showed a decrease of more than 10 folds in the three selected clones compared with MCF-7/ADR and control cells. The MTT assay was used to evaluate the adriamycin cytotoxic effect on the screened stable transfectants and control cells. As depicted in **Figure 1B**, the transfectants of shMDR1/MCF-7/ADR showed significantly different IC_50_ values of adriamycin (the IC_50_ values of clones 1 to 3 were 11.25±2.96, 13.22±3.68, and 2.13±4.72) compared with MCF-7/ADR cells (the IC_50_=34.29±2.47) and control cells (IC_50_=30.80±5.05) (*P*<0.05).

### Down-regulation of P-gp decreased the invasion and migration of MCF-7/ADR cells

**Figure 2:**
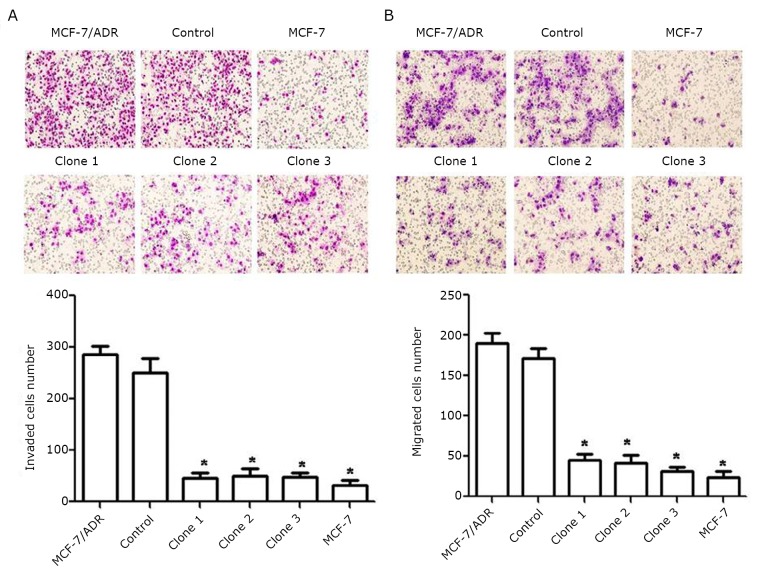
Down-regulation of P-gp decreased the invasion and migration of MCF-7/ADR cells. The invasion (A) and migration (B) assay revealed that the numbers of down-expressed P-gp cells attached to the bottom of the membrane were markedly lower than those of control cells. Each column and bar shows the mean ± SD. The experiments were repeated at least three times. The statistical significance was assessed by one-way ANOVA, *P<0.05 *vs*. control.

As shown in **Figure 2A**, the invasion ability of cells with inhibited P-gp expression was significantly decreased compared with MCF-7/ADR and control cells. Consistently, the migration abilities (**Figure 2B**) of the three clones with stably inhibited P-gp expression were also markedly reduced compared with the parental and control cells.

### Co-immunoprecipitation and co-localization of Anxa2 and P-gp in MCF-7/ADR cells

**Figure 3:**
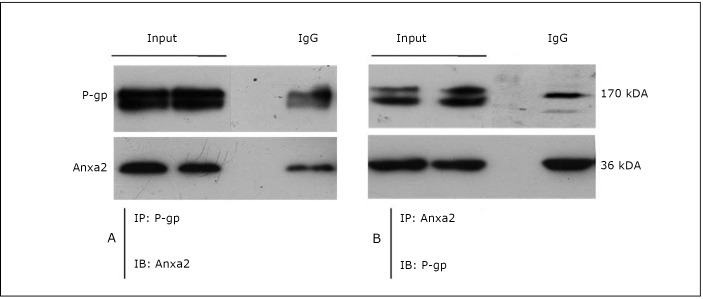
Co-immunoprecipitation of Anxa2 and P-gp in MCF-7/ADR cells. Immunoprecipitation (IP) and immunoblotting (IB). The MCF-7/ADR cell lysate was immunoprecipitated with the indicated antibodies and immunoblotted with corresponding antibodies.

**Figure 4:**
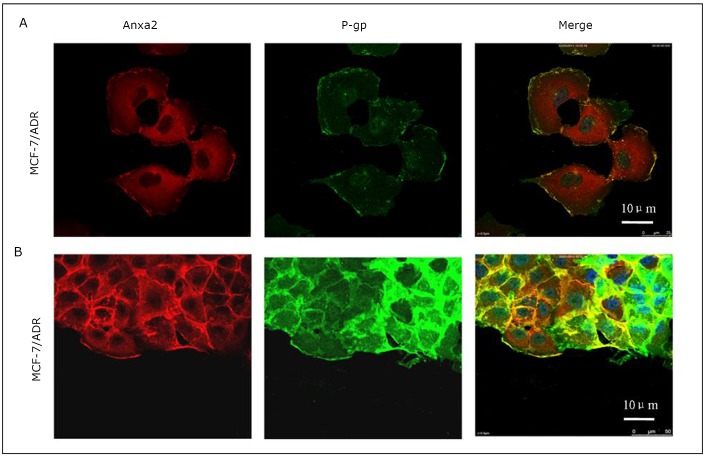
Co-localization of Anxa2 and P-gp in MCF-7/ADR cells. A: Confocal immunofluorescence microscopy analysis showed that P-gp (green) and Anxa2 (red) were co-localized at the cell membrane of MCF-7/ADR cells. B: Wound-healing assay also indicated that P-gp (green) and Anxa2 (red) coexpresssed at the cell membrane of migrating MCF-7/ADR cells along the edge of the “wound”. Scale bar=10 µm.

Co-immunoprecipitation was carried out in MCF-7/ADR whole cell lysates with protein G-Sepharose beads and antibodies against Anxa2 and P-gp. As shown in **Figure 3A**, Anxa2 protein was pulled down by anti-P-gp antibody, and P-gp was also co-immunoprecipitated with Anxa2 by an anti-Anxa2 antibody (**Figure 3B**). The cellular localization of the 2 proteins was also analyzed by confocal microscopy. The double immunoﬂuorescent analysis showed that the expression of P-gp (green) in MCF-7/ADR cells merged with Anxa2 (red), suggesting their physical association with MCF-7/ADR cells (**Figure 4A**). A wound-healing assay obtained similar results. As indicated in **Figure 4B**, P-gp and Anxa2 were co-expressed at the edge of the “wound” of MCF-7/ADR cells.

## Discussion

P-gp is encoded by the MDR1 (ABCB1) gene located in chromosome 7q21.1. P-gp is a broad-spectrum multidrug efflux pump that recognizes a variety of structurally unrelated chemotherapeutic agents, called P-gp substrates^[^^8^^-^^10^^]^. MCF-7/ADR (adriamycin resistance) reportedly displays significantly increased invasion compared with its parental MCF-7 wild-type cells ^[^^1^^, ^^11^^, ^^12^^]^. The present study showed that the down-regulation of P-gp by shMDR1 significantly decreased the rate of MCF-7/ADR cell invasion and migration (**Figure 2**). In addition to regulating MDR, P-gp may possess many properties that can lead to poor prognosis in many forms of malignancies. In recent years, increasing evidence indicates that drug resistance and tumor invasion/metastasis may be functionally linked, although the molecular mechanisms underlying this phenomenon remain largely unknown ^[^^13^^]^. Our previous study demonstrates that the expression of Anxa2 increases when breast cancer MCF-7 cells acquire drug resistance (P-gp-over-expressing adriamycin-resistant breast cancer cells, denoted as MCF-7/ADR) ^[^^7^^]^. Anxa2 may also play essential functions in mediating MDR-induced invasion/metastasis. Recent studies suggest that interactions between Anxa2 and its binding proteins may play important roles in the tumor microenvironment and they act together to promote cancer metastasis ^[^^14^^]^. Interestingly, it was found that P-gp and Anxa2 co-immunoprecipitated with each other in MCF-7/ADR cells (**Figure 3**). P-gp and Anxa2 proteins were also co-localized on the plasma membrane of multidrug-resistant breast cancer cells (**Figure 4**). These interesting findings indicate that the interaction between P-gp and Anxa2 possibly plays an important role in driving the invasion and migration of MDR cancer cells. However, the roles of P-gp and Anxa2 in regulating the migration and invasion of breast cancer cells still need further investigation.

In conclusion, P-gp can promote the invasion and migration of multidrug-resistant breast cancer cells. An interaction between P-gp and Anxa2 is possibly responsible, at least in part, for the association between MDR and invasion/migration potentials in breast cancer cells. The present study may provide foundation for future studies regarding the potential relationships of the MDR phenotype with cancer invasion and metastasis.

## References

[1] Yang JM Xu Z Wu H Overexpression of extracellular matrix metalloproteinase inducer in multidrug resistant cancer cells. Mol Cancer Res 2003; 1: 420-427 12692261

[2] Reimers N Zafrakas K Assmann V Expression of extracellular matrix metalloproteases inducer on micrometastatic and primary mammary carcinoma cells. Clin Cancer Res 2004; 10: 3422-3428 15161697 10.1158/1078-0432.CCR-03-0610

[3] Marieb EA Zoltan-Jones A Li R Emmprin promotes anchorage-independent growth in human mammary carcinoma cells by stimulating hyaluronan production. Cancer Res 2004; 64: 1229-1232 14983875 10.1158/0008-5472.can-03-2832

[4] Miletti-González KE Chen S Muthukumaran N The CD44 receptor interacts with P-glycoprotein to promote cell migration and invasion in cancer. Cancer Res 2005; 65: 6660-6667 16061646 10.1158/0008-5472.CAN-04-3478

[5] Bajorath J Greenfield B Munro SB Identification of CD44 residues important for hyaluronan binding and delineation of the binding site. J Biol Chem 1998; 273: 338-343 9417085 10.1074/jbc.273.1.338

[6] Bosch I Croop J. P-glycoprotein multidrug resistance and cancer. Biochim Biophys Acta 1996; 1288: F37-54 8876632 10.1016/0304-419x(96)00022-4

[7] Zhang F Zhang L Zhang B Anxa2 plays a critical role in enhanced invasiveness of the multidrug resistant human breast cancer cells. J Proteome Res 2009; 8: 5041-5047 19764771 10.1021/pr900461c

[8] Endicott JA Ling V The biochemistry of P-glycoprotein-mediated multidrug resistance. Annu Rev Biochem 1989; 58: 137-171 2570548 10.1146/annurev.bi.58.070189.001033

[9] Gottesman MM Pastan I Biochemistry of multidrug resistance mediated by the multidrug transporter. Annu Rev Biochem 1993; 62: 385-427 8102521 10.1146/annurev.bi.62.070193.002125

[10] Ford JM Hait WN Pharmacology of drugs that alter multidrug resistance in cancer. Pharmacol Rev 1990; 42: 155-199 2217530

[11] Thompson EW Paik S Brunner N Association of increased basement membrane invasiveness with absence of estrogen receptor and expression of vimentin in human breast cancer cell lines. J Cell Physiol 1992; 150: 534-544 1537883 10.1002/jcp.1041500314

[12] dit Faute MA, Laurent L, Ploton D, et al. Distinctive alterations of invasiveness, drug resistance and cell-cell organization in 3D-cultures of MCF-7, a human breast cancer cell line, and its multidrug resistant variant. Clin Exp Metastasis 2002; 19: 161-168. 10.1023/a:1014594825502 11964080

[13] Fletcher JI Haber M Henderson MJ ABC transporters in cancer: more than just drug efflux pumps. Nat Rev Cancer 2010; 10: 147-156 20075923 10.1038/nrc2789

[14] Lokman NA Ween MP Oehler MK The role of annexin A2 in tumorigenesis and cancer progression. Cancer Microenviron 2011; 4: 199-208 21909879 10.1007/s12307-011-0064-9 PMC3170418

